# Comparison of Effectiveness and Safety of Direct-Acting Oral Anticoagulants and Vitamin K Agonists in Patients With Atrial Fibrillation and End-Stage Kidney Disease: A Systematic Review and Meta-Analysis

**DOI:** 10.7759/cureus.57447

**Published:** 2024-04-02

**Authors:** Tanya Sinha, Abshiro H Mayow, Taslova Tahsin Abedin, Chaw N Phoo, Saima H Shawl, Ali Muhammad, Samer Kholoki, Shamsha Hirani

**Affiliations:** 1 Medicine, Tribhuvan University, Kathmandu, NPL; 2 Medicine, St. Georges University School of Medicine, Chicago, USA; 3 Medicine, Sylhet MAG Osmani Medical College, Hyattsville, USA; 4 Internal Medicine, University of Medicine, Mandalay, Mandalay, MMR; 5 Internal Medicine/Sleep Medicine, Midwest Sleep and Wellness Clinic, Chicago, USA; 6 Medicine, Chattogram Medical College Hospital, Chittagong, BGD; 7 Neurology, King Edward Medical Univeristy, Lahore, Lahore, PAK; 8 Medicine, King Edward Medical Univeristy, Lahore, Lahore, PAK; 9 Internal Medicine, La Grange Memorial Hospital, Chicago, USA; 10 Cardiology, Baqai Hospital, Karachi, PAK

**Keywords:** systematic review and meta-analysis, end stage renal disease, atrial fibrillation, warfarin, direct-acting oral anti-coagulants

## Abstract

The objective of the study is mentioned, but it could be further clarified by explicitly stating the aim to compare the effectiveness and safety of direct oral anticoagulants (DOACs) versus vitamin K antagonists (VKAs) specifically in patients with atrial fibrillation (AF) and end-stage renal disease (ESRD). We conducted a thorough electronic search of the literature, encompassing databases such as PubMed, EMBASE, Cochrane Library, and Web of Science from their inception up to March 5, 2024. Furthermore, we meticulously examined the bibliographies of included studies to identify additional relevant literature. The reporting of this meta-analysis adhered to the guidelines outlined in the Preferred Reporting of Systematic Review and Meta-analysis guidelines. The endpoints evaluated in this meta-analysis included all-cause mortality, stroke or systemic embolism, and major bleeding. Data analysis was carried out utilizing RevMan Version 5.4 (Cochrane, London, United Kingdom). Dichotomous outcomes, including all-cause mortality, stroke or systemic embolism, and major bleeding, were presented as risk ratios (RRs) with corresponding 95% confidence intervals (CI). A total of 11 studies were incorporated in this meta-analysis, comprising a pooled sample size of 44,863 participants with AF. The pooled analysis revealed no significant disparity between DOACs and VKAs concerning stroke or systemic embolism (RR: 0.93, 95% CI: 0.77 to 1.14) and all-cause mortality (RR: 0.86, 95% CI: 0.74 to 1.00). However, there was a noteworthy reduction in the risk of major bleeding events associated with DOACs compared to VKAs (RR: 0.84, 95% CI: 0.73 to 0.96). Consequently, DOACs may be considered a viable alternative to warfarin in patients with ESRD. However, we need further larger clinical trials to validate these findings.

## Introduction and background

Atrial fibrillation (AF) is recognized as the prevailing sustained arrhythmia, with an approximate prevalence in the general populace ranging from 0.5% to 1% [1,2]. In the management of patients with AF, stroke prevention remains pivotal, and since 2009, direct oral anticoagulants (DOACs) have emerged as a viable, safe, and convenient alternative to vitamin K antagonists (VKAs). The widespread adoption of DOACs has been bolstered by robust randomized trial data, which demonstrate comparable or even superior efficacy and safety profiles compared to warfarin [3]. Patients with chronic kidney disease (CKD) face notably heightened risks of venous thromboembolism (VTE) [4] and stroke [5], regardless of the extent of renal impairment, a risk further exacerbated in those with end-stage kidney disease (ESKD) undergoing dialysis. Moreover, individuals within this susceptible demographic encounter a heightened likelihood of bleeding, which may arise from complications associated with renal disease like anemia, thrombocytopenia, and secondary hyperparathyroidism. Additionally, bleeding risk may be exacerbated by concurrent administration of medications such as anticoagulants and antiplatelets, or by the presence of specific coagulopathies [6].

While VKAs are usually prescribed for patients with normal or mildly impaired renal function, their use for stroke prevention in AF patients undergoing dialysis remains contentious. Notably, most studies have excluded dialysis patients, making the assessment of the suitability of DOACs in this particular demographic complicated [7]. Moreover, current evidence indicates that in individuals with impaired renal function, warfarin may not confer the same degree of reduction in thromboembolic risk as observed in those without CKD [8]. Consequently, the dearth of robust evidence supporting either class of oral anticoagulants in the dialysis population has led to significant practice variation and uncertainty among physicians.

A recent systematic review revealed that, when compared to warfarin, apixaban did not demonstrate a significant decrease in the incidence of VTE recurrence among ESKD patients undergoing dialysis. However, fewer instances of major bleeding were noted in patients treated with apixaban [9]. Regarding stroke prevention in non-valvular atrial fibrillation (NVAF) patients, a separate systematic review revealed that while apixaban, given at a standard dosage of 5 mg twice daily, reduced the risk of stroke and systemic embolism in comparison to warfarin, this dosage was associated with increased bleeding rates [10]. Given the numerous studies conducted since the last meta-analysis assessing the efficacy and safety of DOACs in ESKD patients, we are currently undertaking an updated systematic review and meta-analysis to compare the efficacy and safety of DOACs and VKAs in individuals with AF and end-stage renal disease (ESRD).

## Review

Methodology

We systematically conducted an electronic literature search encompassing the PubMed, EMBASE, Cochrane Library, and Web of Science databases from their inception up to March 5, 2024. Additionally, we scrutinized the bibliographies of the included studies to identify supplementary literature. We investigated conference proceedings to identify relevant conference papers for our study. Our search strategy incorporated the terms "direct oral anticoagulants," "vitamin K antagonist", "atrial fibrillation" and "end-stage kidney disease" along with their synonyms and corresponding medical subject heading (MeSH) terms, without language restrictions, to retrieve articles pertinent to this investigation. Two authors independently performed the search, and any disparities between them were resolved through consensus or consultation with a third author. This meta-analysis was reported as per the guidelines of Preferred Reporting of Systematic Review and Meta-analysis (PRISMA) guidelines.

Inclusion and Exclusion Criteria

We employed the Population, Intervention, Comparison, Outcomes, and Study Design (PICOS) model to determine study eligibility. The targeted population comprised individuals with AF and end-stage CKD (GFR < 15 mL/min/1.73 m^2^). The intervention group consisted of patients receiving DOACs, whereas the comparison group comprised patients receiving warfarin. Outcomes evaluated in this meta-analysis encompassed stroke, major bleeding, and all-cause mortality. We included both randomized controlled trials (RCTs) and observational studies. Studies involving patients with a GFR >15 mL/min/1.73 m^2^ were excluded from the analysis to exclude patients without ESRD. Additionally, studies lacking VKAs as the control group, animal studies, case reports, editorials, expert opinions, and unpublished studies were excluded.

Selection of Studies and Data Collection

Titles, abstracts, and full-text articles underwent screening for inclusion. Data abstracted from selected articles encompassed: 1) name of the first author and publication year; 2) study design 3) characteristics of the population; 4) country of study conduct; 5) type of DOAC utilized; 6) sample size; 7) follow-up duration and 8) endpoints of assessment, including mortality, stroke or systemic embolism, and major bleeding. These data were independently collected by two authors utilizing data abstraction forms. Discrepancies were resolved through consensus during meetings.

Data Analysis

We conducted data analysis using RevMan Version 5.4 (Cochrane, London, United Kingdom). Dichotomous outcomes, such as all-cause mortality, stroke or systemic embolism, and major bleeding, were presented as risk ratios (RRs) with 95% confidence intervals (CI). A significance level of p < 0.05 was applied. Forest plots were generated for each assessed outcome to illustrate the aggregated findings. Heterogeneity was evaluated using I-square statistics, where an I-square value exceeding 50% was considered indicative of significant heterogeneity. To address potential heterogeneity stemming from variations in study drugs, doses, population characteristics, and study design, a random-effect model was employed to compute pooled estimates.

Results

**Figure 1:**
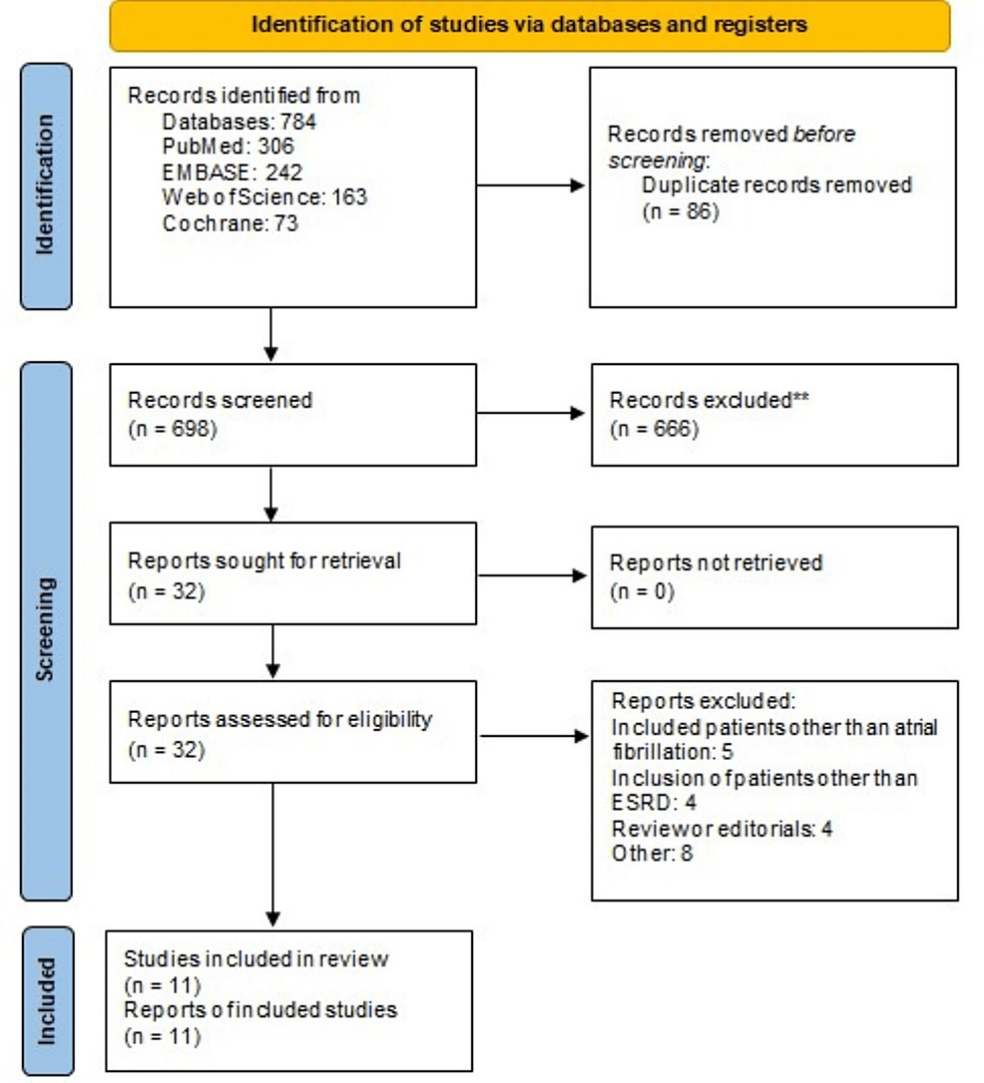
PRISMA flowchart of study selection

Through systematic database searching, a total of 784 studies were identified. After eliminating duplicate records, 698 unique studies remained for initial screening, which was followed by a meticulous full-text assessment of eligibility criteria. Ultimately, 11 studies met the inclusion criteria and were selected for inclusion in this meta-analysis, encompassing a pooled sample size of 44,863 participants. The study selection process is visually represented in Figure 1. Table 1 shows the characteristics of the included studies. Among the included studies, three were RCTs. The majority of studies, totaling five, were conducted in the United States. Apixaban was the most frequently utilized DOAC in the included studies. The duration of follow-up across the included studies ranged from 6 to 60 months.

**Table 1:** Characteristics of included studies DOAC: Direct oral anticoagulant; VKA: Vitamin K antagonist; RCT: Randomized-control trial; NS: Not specify

Author	Year	Study Design	Region	Groups	Types of DOAC	Sample Size	Follow-up	Age (Years)	Males (n)	Diabetes (n)	Hypertension (n)
Chan et al. [11]	2016	Retrospective	United States	DOAC	Rivaroxaban/dabigartan	525	24 months	67.7	312	363	451
				VKA		8064		70.6	4935	5475	7137
Lara et al. [12]	2023	Retrospective	United States	DOAC	Apixaban	10037	60 months	NS	NS	NS	NS
				VKA		10037					
Lin et al. [13]	2021	Retrospective	Japan	DOAC	Rivaroxaban	173	27.4 months	69	74	88	135
				VKA		3185		69	1561	1593	2484
Moore et al. [14]	2023	Retrospective	United States	DOAC	Apixaban	53	24 months	NS	NS	NS	NS
				VKA		57					
Noseworthy et al. [15]	2019	Retrospective	United States	DOAC	Dabigartan, rivaroxaban and apixaban	568	6.72 months	NS	NS	NS	NS
				VKA		859					
Pokomey et al. [16]	2022	RCT	United Kingdom	DOAC	Apixaban	82	12 months	69	48	42	79
				VKA		72		68	42	47	67
Reinecke et al. [17]	2023	RCT	Germany	DOAC	Apixaban	48	>12 months	74.7	37	NS	NS
				VKA		49		74.8	37		
See et al. [18]	2021	Retrospective	Taiwan	DOAC	Dabigartan, rivaroxaban and apixaban	448	60 months	74.3	222	276	371
				Warfarin		448		75.2	213	285	362
Siontis et al. [19]	2018	Retrospective	United States	DOAC	Apixaban	2351	17 months	NS	NS	NS	NS
				Warfarin		7053					
Vriese et al. [20]	2020	RCT	Belgium	DOAC	Rivaroxaban	46	18 months	79.9	35	20	NS
				VKA		44		80.3	25	20	
Welander et al. [21]	2023	Retrospective	Sweden	DOAC	Dabigartan, rivaroxaban and apixaban	83	48 months	NS	NS	NS	NS
				VKA		581					

Meta-Analysis of Outcomes

**Figure 2:**
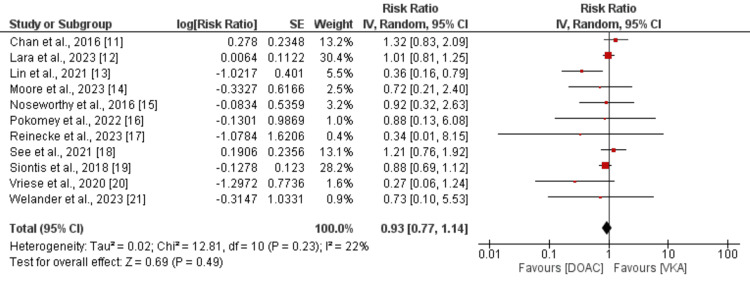
Comparison of stroke or systemic embolism between DOAC and VKA DOAC: Direct oral anticoagulant; VKA: Vitamin K antagonist Reference: [11-21]

**Figure 3:**
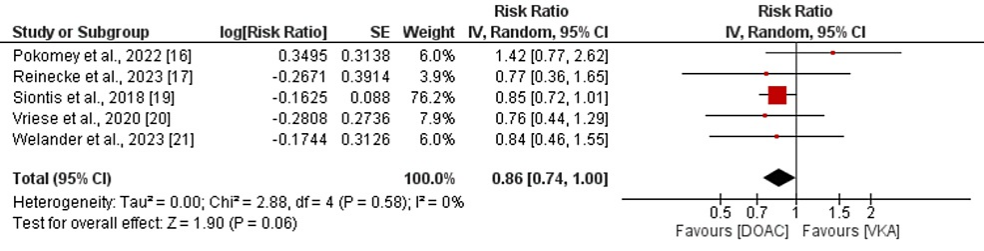
Comparison of all-cause mortality between DOAC and VKA DOAC: Direct oral anticoagulant; VKA: Vitamin K antagonist References: [16-17,19-21]

Eleven studies compared the risk of stroke or systemic embolism between DOAC and VKA in patients with AF. As shown in Figure 2, the risk of stroke or systemic embolism was not significantly different between DOAC and VKA (RR: 0.93, 95% CI: 0.77 to 1.14). No significant heterogeneity was reported among the study results (I-square: 22%). All-cause mortality between the two groups was compared by 5 of the 11 included studies with a pooled sample size of 10419 patients. As shown in Figure 3, the risk of all-cause mortality was 14% lower in patients receiving DOAC compared to warfarin. However, the difference was statistically insignificant (RR: 0.86, 95% CI: 0.74 to 1.00). No significant heterogeneity was reported among the study results (I-square: 0%).

**Figure 4:**
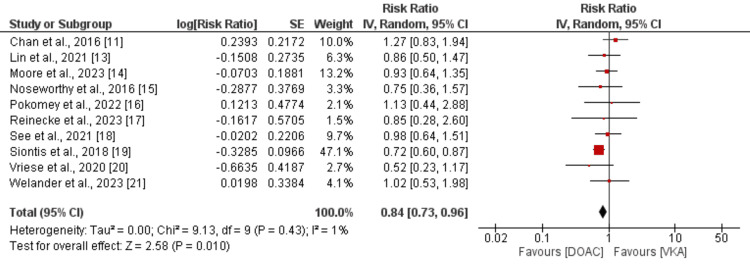
Comparison of major bleeding events between DOAC and VKA DOAC: Direct oral anticoagulants; VKA: Vitamin K antagonist References: [11-21]

For safety analysis, we compared major bleeding events between patients receiving DOAC and VKA in patients with AF. The forest plot for this comparison is shown in Figure 4. As shown in Figure 4, the risk of major bleeding events was 16% lower in patients receiving DOAC compared to VKA and the difference was statistically insignificant (RR: 0.84, 95% CI: 0.73 to 0.96). No significant heterogeneity was reported among the study results (I-square: 1%).

Discussion

This meta-analysis investigated the efficacy and safety of DOACs versus VKAs in patients with AF and ESRD. The study findings indicated that DOACs carry a comparable risk to VKAs regarding the composite outcome of ischemic stroke or systemic embolism, as well as all-cause mortality. Nonetheless, individuals treated with DOACs exhibited a markedly reduced likelihood of experiencing major bleeding events compared to those receiving VKAs. This meta-analysis amalgamated data from both observational and RCT studies, representing the latest comprehensive comparison between DOACs and VKAs in AF patients with ESRD.

Prior research has underscored the established advantages of DOACs over warfarin in individuals with NVAF [22]. Nevertheless, the presence of ESRD complicates the intricate dynamics of bleeding and coagulation pathways, leading to the exclusion of such patients from major DOAC clinical trials. Notably, the meta-analysis conducted by Abdullah et al. [23] found that apixaban is linked to a reduced risk of major bleeding and a comparable risk of stroke compared to warfarin in AF patients with ESRD. Diverging from previous meta-analyses, our study exclusively incorporated research conducted on AF patients and also integrated recently conducted observational studies.

Although warfarin offers favorable pharmacodynamic characteristics for patients with ESRD due to its hepatic elimination and resistance to dialysis, its unpredictable dose-response relationship may predispose patients to bleeding incidents. In addition to its narrow therapeutic window and the need for frequent international normalized ratio monitoring, warfarin contributes to arterial vascular calcification [24]. This phenomenon is thought to occur through the inhibition of the vitamin K-dependent enzyme matrix gamma-carboxyglutamate Gla protein, which typically inhibits calcification processes [25]. The findings of the present meta-analysis confirm a heightened risk of bleeding in patients receiving VKA.

The incidence of bleeding in patients undergoing dialysis is heightened with warfarin, potentially attributed to platelet dysfunction. Platelet dysfunction stems from intrinsic platelet irregularities as well as compromised platelet-vessel wall interactions. Patients with renal failure exhibit deficiencies across the classic stages of platelet response to injury, including activation, recruitment, adhesion, and aggregation. While dialysis may mitigate these deficiencies to some extent, complete correction remains elusive. Moreover, the dialysis procedure itself may exacerbate bleeding tendencies. Hemodialysis is additionally linked to thrombotic events due to chronic platelet activation induced by contact with artificial surfaces during the procedure [26]. Notably, the use of warfarin has not demonstrated significant efficacy in reducing stroke and mortality rates and has been associated with an elevated risk of major bleeding, as evidenced by previous meta-analyses [27]. Similarly, in a single-center retrospective cohort study conducted by Sarratt et al., comparisons between apixaban and warfarin in patients with AF undergoing hemodialysis revealed no significant disparities in the rates of major bleeding, clinically relevant non-major bleeding, or minor bleeding between the two treatment groups [28].

One of the primary advantages of DOACs compared to warfarin is the absence of necessity for routine laboratory monitoring. However, in specific patient subgroups such as those undergoing dialysis, there may be a need to determine either the quantitative DOAC concentration or its qualitative effect. None of the studies included in our analysis evaluated the levels or effects of DOACs, which may mirror real-world practices regarding DOAC monitoring. Unlike apixaban and edoxaban, which are cleared by dialysis at rates of 6% and 9%, respectively, dabigatran exhibits a clearance of 50%-60% within four hours of hemodialysis. Data on rivaroxaban clearance through dialysis remains unpublished. These findings elucidate why apixaban was predominantly utilized in the studies included in our analysis [29].

The ongoing trials, namely the German AF network, and The Strategies for the Management of Atrial Fibrillation in patiEnts Receiving Dialysis (SAFE-D) trial are currently evaluating the comparison between DOACs and warfarin in patients diagnosed with AF and ESRD [30,31]. Given that the majority of the studies investigating these medications have an observational design, these trials hold significant promise in informing the development of anticoagulation guidelines for this specific patient population.

Clinical Implications

The study findings suggest that DOACs offer a promising alternative to VKAs in managing anticoagulation for patients with AF and ESRD. With DOACs showing comparable efficacy in preventing ischemic stroke or systemic embolism and a lower risk of major bleeding compared to VKAs, clinicians may consider DOACs as preferred agents in this population. However, further research, particularly from ongoing trials, is crucial to establish clearer guidelines for anticoagulation management in patients with AF and ESRD.

Study Limitations

The study's findings are subject to several limitations that may impact the robustness and generalizability of the conclusions. The majority of included studies adopted an observational design, introducing inherent biases and limitations such as confounding variables and selection bias. Variability in endpoint definitions, particularly concerning safety and efficacy outcomes, further complicates interpretation. Additionally, the use of different DOAC formulations with varied dosages across studies adds complexity and may hinder direct comparisons. Heterogeneous inclusion and exclusion criteria, alongside disparate definitions of outcomes and variations in follow-up duration, limit the ability to draw definitive conclusions. Subgroup analysis based on specific DOACs was challenging due to the predominant use of apixaban and the lack of outcome specificity for other DOACs. Last, retrospective studies faced challenges in accurately identifying patients receiving concomitant antiplatelet therapy, potentially influencing results. Despite efforts to address these limitations, further research is warranted to validate findings and refine anticoagulation guidelines for patients with AF and ESRD.

## Conclusions

In conclusion, our meta-analysis compared the efficacy and safety of DOACs versus VKAs in patients with AF. Although no significant difference was observed in the risk of stroke or systemic embolism between DOACs and VKAs, DOACs showed a trend toward lower all-cause mortality and major bleeding events. It's noteworthy that the included studies predominantly had an observational design, introducing limitations in endpoint definitions, drug variations, and patient characteristics. Therefore, further studies, including RCTs, are needed to validate these findings and address specific research questions. Future studies should focus on comparing the efficacy and safety of specific DOACs versus VKAs in patients with AF and ESRD, providing clearer insights for clinical decision-making. Additionally, future studies should also focus on finding the optimal dosing of these drugs to guide clinicians about maximizing therapeutic benefits while minimizing adverse effects.
